# Generation of a Felinized Swine Endothelial Cell Line by Expression of Feline Decay-Accelerating Factor

**DOI:** 10.1371/journal.pone.0117682

**Published:** 2015-02-11

**Authors:** Luna Izuhara, Norifumi Tatsumi, Shuji Miyagawa, Satomi Iwai, Masahito Watanabe, Shuichiro Yamanaka, Yuichi Katsuoka, Hiroshi Nagashima, Hirotaka J. Okano, Takashi Yokoo

**Affiliations:** 1 Division of Regenerative Medicine, The Jikei University School of Medicine, Tokyo, Japan; 2 Department of Anatomy, The Jikei University School of Medicine, Tokyo, Japan; 3 Division of Organ Transplantation, Department of Regenerative Medicine, Osaka University Graduate School of Medicine, Osaka, Japan; 4 Laboratory of Small Animal Surgery I, School of Veterinary Medicine, Kitasato University, Aomori, Japan; 5 Meiji University International Institute for Bio-Resource Research, Kawasaki, Japan; 6 Division of Nephrology and Hypertension, Department of Internal Medicine, The Jikei University School of Medicine, Tokyo, Japan; University of Tokushima, JAPAN

## Abstract

Embryonic stem cell research has facilitated the generation of many cell types for the production of tissues and organs for both humans and companion animals. Because ≥30% of pet cats suffer from chronic kidney disease (CKD), xenotransplantation between pigs and cats has been studied. For a successful pig to cat xenotransplant, the immune reaction must be overcome, especially hyperacute rejection. In this study, we isolated the gene for feline decay-accelerating factor (fDAF), an inhibitor of complement proteins, and transfected a swine endothelial cell line with *fDAF* to “felinize” the pig cells. These fDAF-expressing cells were resistant to feline serum containing anti-pig antibodies, suggesting that felinized pig cells were resistant to hyperacute rejection. Our results suggest that a “felinized” pig kidney can be generated for the treatment of CKD in cats in the future.

## Introduction

It is anticipated that regenerative medicine and xenotransplantation will provide new therapies for people awaiting donor organs. We previously demonstrated generation of self-organs from autologous mesenchymal stem cells (MSCs) using the inherent developmental system of a xenogeneic host [1,2]. The growth of human MSCs at a specific organ location in a whole-embryo culture allows these cells to commit to the cellular fate of that organ. Using this approach, we expect to be able to develop chimeric kidneys. Our xenotransplantation model involves erythropoietin (EPO)-producing cells that differentiate from the host cells in the transplanted metanephros [3,4]. This research may be clinically utilized to combat human kidney diseases, but the level of EPO production is far below that required for therapeutic efficacy. Further studies using a larger animal, such as the pig, are required.

Regenerative medicine, such as the production of EPO-expressing chimeric kidneys, will probably be applied not only to humans but also to companion animals. A public survey in 2010 by the Cabinet Office of the Japanese Government revealed that 72.5% of the Japanese population owned a pet and that the breeding rate of cats was 30.9% (http://www8.cao.go.jp/survey/h22/h22-doubutu/2-1.html). However, at least 30% of pet cats suffer from chronic kidney disease (CKD) [5], and most cats with CKD do not survive the complications associated with renal anemia [6]. Recombinant human EPO (rhEPO) is an effective treatment for human CKD [7]. rhEPO is also effective in the treatment of feline CKD, but after a few weeks, anti-EPO antibodies are produced; thus, this therapy is only transiently effective in cats [8]. It has also been reported that the use of recombinant feline EPO (fEPO) has the same effects as rhEPO [9]. Therefore, we transplanted a metanephros to induce the differentiation of autologous stem cells into EPO-producing cells. Our preliminary experiments involving the transplantation of a pig metanephros into the cat omentum resulted in the production of fEPO [10]. However, four of the six cats had pre-existing anti-pig antibodies, which caused the hyperacute rejection and destruction of the xenotransplant before its development (unpublished data). Therefore, to apply our system to all cats, we must prevent this hyperacute rejection during xenotransplantation between pigs and cats. Hyperacute rejection is caused by the binding of xenoreactive antibodies to donor vascular endothelial cells after the activation of the recipient’s complement response [11]. Donor decay-accelerating factor (DAF, CD55)-transfected cells were established to prevent the activation of the recipient’s complement proteins [12], and human DAF-expressing pigs were produced transgenically and have been used as “humanized” pigs in xenotransplantation between pigs and humans [13–17].

DAF is a glycosylphosphatidylinositol (GPI)-anchored membrane inhibitor of complement proteins and inhibits complement activation by interfering with the function of C3 and C5 convertases in both the classical and alternative pathways [18,19].

In this study, we cloned feline *DAF* (*fDAF*) and used it to transfect a swine endothelial cell line to “felinize” it. These cells were stimulated with feline serum containing anti-pig antibodies. Using a lactate dehydrogenase (LDH) assay, we found that the fDAF-expressing cells were resistant to feline serum. These results suggest that fDAF-expressing cells may be resistant to hyperacute rejection. Our results suggest a possible strategy for the medical treatment of feline CKD using xenotransplantation between cats (with renal insufficiency) and pigs.

## Materials and Methods

### Animals and ethics statement

Specific pathogen-free domestic cats (*Felis silvestris catus*; Nisseiken, Tokyo, Japan) were used in this study. Blood samples were obtained from the cats’ medial saphenous vein. All animal experiments were approved by the Animal Experiment Committee of the Jikei University School of Medicine (Decision #21–014).

### Cloning of *fDAF*

Total RNA was isolated from leukocytes from the blood samples of cats. First-strand cDNA was then synthesized using the PrimeScript II 1st strand cDNA Synthesis Kit (Takara Bio Inc., Ohtsu, Japan) to clone the partial gene sequence using the SMART RACE cDNA Amplification Kit (Clontech, CA, USA) for 3′ rapid amplification of cDNA ends (RACE). Two sets of primers (5′-GCGTTTTCTTGGCGAGCTAA-3′ and 5′-AGGAGCTGATGGGACTTTCGT-3′ and 5′-TGCATCGCTCAAGAGGTCTTT-3′ and 5′-TTTTCCGTGGATTTTGAATGG-3′) were used to confirm the partial sequence, excluding the 3′ sequence from Ensemble (sequence ID: ENSFCAG00000007875). A partial sequence of *fDAF* was amplified by PCR using Blend Taq Plus DNA polymerase (Toyobo, Osaka, Japan) and then ligated into the pGEM-T Easy vector (Promega, WI, USA) for sequencing. Based on the sequence data, we designed primers for 3′ RACE. PCR was performed with the primers 5′-GGAGAATGGAGTGGCCTGCCCCCTG-3′ and UPM (Clontech), and then the PCR product was reamplified. Following this, the PCR product was ligated into the pGEM-T Easy vector, and four independent clones were sequenced; thus, we identified the 3′ sequence including the polyA sequence. To clone the 5′ fragment, including the start sequence of *fDAF*, primers (5′-ATGGGTCCCGCGCGGCGGA-3′ and 5′-TCAGGGGGCAGGCCACTCCA-3′) were designed on the basis of the Ensemble sequence. PCR was performed using Ex Taq DNA polymerase, and the PCR product was then ligated into the pGEM-T Easy vector. Four independent clones were sequenced, and we identified the 5′ sequence downstream of the start codon. Both DAF 5′ and DAF 3′ were digested with *Nco*I and *Kpn*I (Takara), and then the 5′ product was ligated to the 3′ sequence to produce the full-length *fDAF* sequence (GenBank accession number: AB773827).

### Analysis of *fDAF*

A homology search of human DAF (hDAF; NP_000565), mouse DAF (mDAF; NP_034146), and swine DAF (sDAF; NP_998980) with *fDAF* was performed using NCBI’s BLAST. To predict the GPI anchor domain, we used GPI Modification Site Prediction [20–23].

### Cell culture

The swine endothelial cell (sEC) line MYP30 [24] (gifted by Dr. Miyagawa) was cultured in Dulbecco’s modified Eagle’s medium (DMEM; Life Technologies, NY, USA) containing 10% fetal bovine serum (FBS; Gibco, NY, USA), l-glutamine (Gibco), and penicillin/streptomycin (Gibco). The cultures were maintained at 37°C in a humidified atmosphere containing 5% CO_2_.

### Establishment of an fDAF-expressing sEC clone

The expression vector was based on the pCX-EGFP plasmid [25]. Briefly, *fDAF* with the Kozak sequence was amplified by PCR from the plasmid containing the full-length *fDAF* sequence using the following primers: 5′-TTTTGGCAAAGAATTCGCCACCATGGGTCCCGCGCGGCGGAG-3′ and 5′-CCTGAGGAGTGAATTCACTAGTGATTCGGCTAAGTCAG-3′. The amplified product was inserted into the *Eco*RI sites of pCX-EGFP digested with *Eco*RI, which removed the enhanced green fluorescent protein (EGFP) sequence. For antibiotic selection, the puromycin N-acetyltransferase gene under the control of the SV40 promoter was amplified, and this DNA fragment was inserted into the *Hin*dIII site of the constructed vector to generate pCX-fDAF-puroR. Finally, we sequenced the constructed *fDAF* expression vector. The transgene was excised from the plasmid by digestion with *Sal*I and *Bam*HI and then used to transfect the sEC line using the Neon Transfection system (Invitrogen, CA, USA) under the following conditions: pulse voltage, 1100 V; pulse width, 30 ms; pulse number, 1 (program #6). Three days after the electroporation, the transfectants were selected on 2 g/mL puromycin (Life Technologies) for 12 days to isolate puromycin-resistant cells. After drug selection, the puromycin-resistant cells were collected, expanded, and used for the subsequent experiments. In order to check whether *fDAF* transduced cells maintain their characteristic as endothelial cells, we synthesized first-strand cDNA from both the MYP30 sEC line and the fDAF-transfected sEC clone using a PrimeScript II 1st strand cDNA Synthesis Kit (Takara Bio Inc.). A PCR reaction was performed using Blend Taq Plus DNA polymerase. The PCR program included a denaturation step (95°C for 15 s), an annealing step (55°C for 15 s), and an extension step (72°C for 1 min) followed by 30 cycles for ß-actin and 35 cycles for CD31 and VE-cadherin. We used the primers (5′-CTCTTCCAGCCCTCCTTCCT-3′ and 5′-CGACGTCGCACTTCATGATG-3′) for ß-actin (XM_003124280); (5′-CATTTCCAAAGTCAGCAGCA-3′ and 5′-CATCATCATGCCTCCCTTCT-3′) for CD31 (NM_213907); and (5′-CGACTCATCCGACTCTGACA-3′ and 5′-TTTGTGAGTAGCCGTTGCTG-3′) for VE-cadherin (AB046120).

### Evaluation of the specificity of the anti-DAF antibody

PCR was performed to construct the *fDAF-FLAG* expression vector, and the following primers were used: 5′-GAATTCGCCACCATGGGTCCCGCGCGGCGGAG-3′ and 5′-GAATTCTTACTTGTCATCGTCATCCTTGTAGTCGATGTCATGATCTTTATAATCACCGTCATGGTCTTTGTAGTCGCCAATGGTTACTAGCGTCA-3′ for attachment of a FLAG tag to full-length *fDAF*. The PCR product was subcloned into the pGEM-T Easy vector and sequenced. The subcloned fDAF-FLAG was digested with *Eco*RI and inserted into an *Eco*RI site of pCX-puro. The pCX-fDAF-FLAG or pCX (as a mock control) was transfected into 5 × 10^5^ HEK-293 cells using Lipofectamine 2000 according to the manufacturer’s instructions (Invitrogen, Carlsbad, CA). The cells were cultured for 48 h in DMEM containing 20% FBS. Both cell clones were harvested and proteins were extracted with lysis buffer (10 mM Tris-HCl pH 7.2, 150 mM NaCl, 1% Nonidet P-40, 0.05% SDS) containing a protease inhibitor cocktail (Roche; Mannheim, Germany). The proteins and markers (*Precision Plus Protein*; Bio-Rad, CA, USA) were subjected to SDS-PAGE (Bio-Rad) and then transferred to a polyvinylidene difluoride (PVDF) membrane (Millipore, MA, USA). The membrane was blocked with the PVDF Blocking Reagent (Toyobo) and washed in Tris-buffered saline containing 0.1% Tween 20 (TBST) for 60 min at room temperature. The membrane was then incubated with an anti-DAF (i.e., anti-CD55) antibody (1:1000 dilution; HPA024386, Atlas Antibodies, Stockholm, Sweden) overnight at 4°C. The membrane was washed five times with TBST and incubated with a horseradish peroxidase (HRP)-conjugated goat anti-rabbit IgG antibody (1:10000, Nichirei Bioscience Inc., Tokyo, Japan) for 1 h and washed five times with TBST. The ImmunoStar LD (Wako, Osaka, Japan) was used to detect the signals by means of the ChemiDoc XRS+ System with Image Lab (Bio-Rad). The membrane was stripped and reprobed with other antibodies: an anti-DDDDK (anti-FLAG) antibody (1:10000, MBL, Nagoya, Japan) or anti-β-actin antibody (1:2000, Sigma—Aldrich, MO, USA). An HRP-conjugated goat anti-mouse IgG antibody (1:10000, Millipore) served as a secondary antibody. Protein detection was conducted using the same method as described above. For absorption analysis, the anti-DAF antibody was mixed with human DAF protein (Sino Biological Inc., Beijing, China) at the ratio of 1 mol to 20 mol in PBS containing 1% BSA. The solution was mixed on a rotator overnight and then used as a primary antibody.

### Identification of fDAF expression in the *fDAF*-transfected cells

For this immunohistochemical analysis, both the transfected cells and control cells were cultured on collagen-coated coverslips for 24 h and then fixed with 4% paraformaldehyde in PBS. The cells were then pretreated with an antigen retrieval solution (Histo VT One; Nacalai Tesque, Kyoto, Japan) for 20 min at 70°C. The cells were incubated overnight with an anti-CD55 (anti-DAF) antibody (diluted 1:50; HPA024386; Atlas Antibodies) in a humidified chamber at 4°C and then with a biotinylated goat anti-rabbit IgG antibody (1:200; Vector Laboratories, CA, USA) for 1 h at room temperature. To detect the signals, the cells were incubated with Alexa Fluor 488–streptavidin (diluted 1:500; Molecular Probes Invitrogen). The nuclei were counterstained with DAPI (1:10000; Nacalai Tesque). The untransfected cells served as a negative control. For western blot analysis of the fDAF expression in the *fDAF*-transfected cells, both the transfected cells and control cells were cultured on 10-cm dishes under the above conditions. Densitometry of protein bands was performed in the ImageJ software (NIH, USA).

### LDH assay

This assay was performed as a modified version of the method used by Korzeniewski and Callewaert [24,26] using a Cytotoxicity Detection Kit (Roche). The transfected cells were seeded at 2 × 10^4^/well on 96-well plates 1 day before the assay. The cell culture was maintained at 37°C in the humidified atmosphere containing 5% CO_2_ [27]. The next day, the cells were washed twice with serum-free DMEM to remove LDH present in FBS and then incubated in 40% or 80% feline serum diluted with DMEM. The released LDH was measured after 2, 4, and 6 h. The percentage of cytotoxicity was calculated using the following formula:

$$\mathrm{cytotoxicity}\;(\%)\;=\frac{(A-B)-(C-D),}{E-D}$$

where A is the test substance (feline serum) at the maximum concentration used in the experiment, B is the LDH activity of feline serum (background control), C is the spontaneous release of LDH from the sEC line incubated in the absence of feline serum, D is the assay medium only, and E is the maximal release of LDH determined by the addition of 1% Triton X-100. The LDH assay for each time point and serum concentration was independently performed five times. The data were analyzed statistically using Student’s *t* test, and the significance level was set at *P* = 0.05.

## Results

### Cloning of *fDAF*

**Fig 1:**
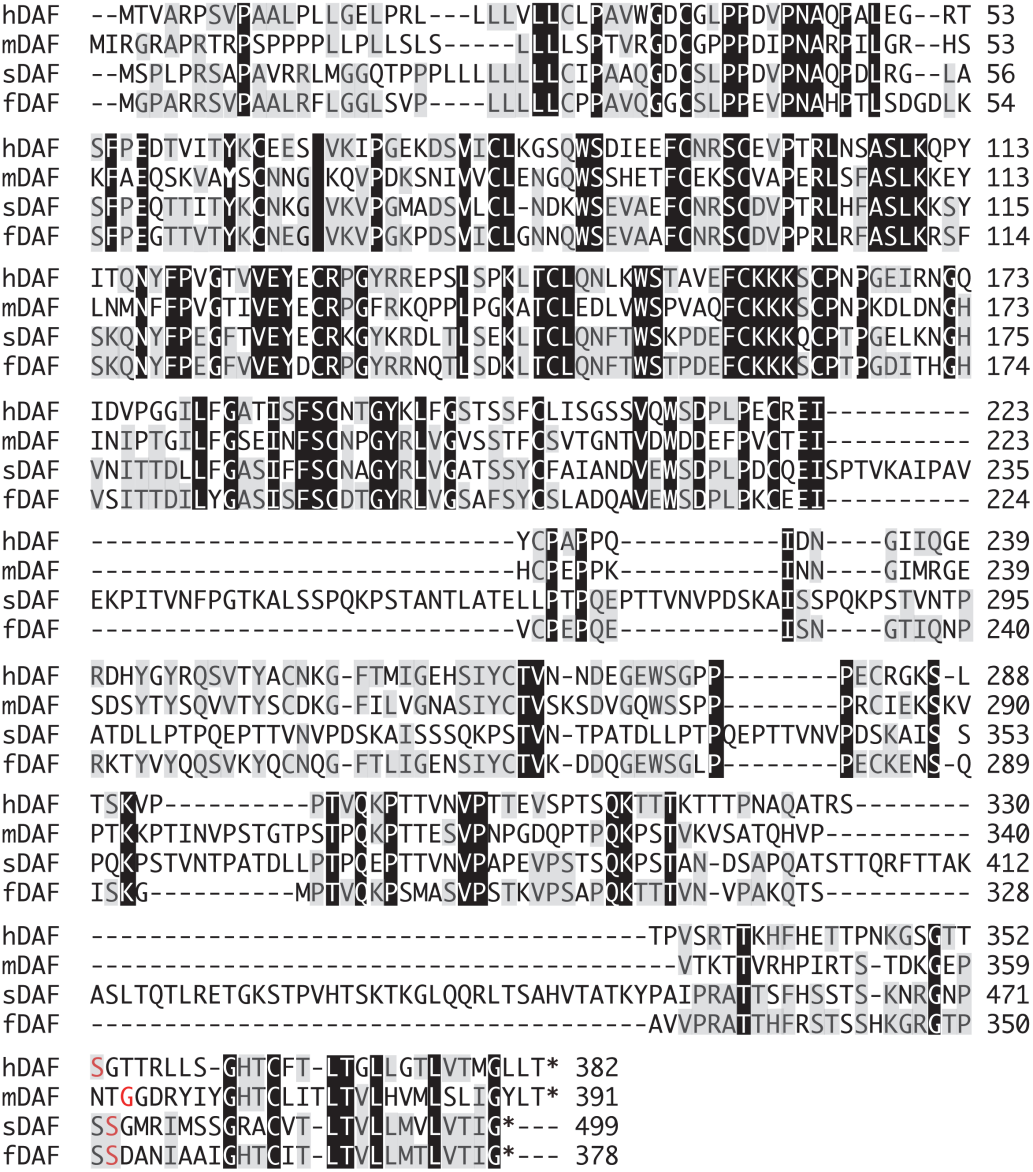
The whole amino acid sequence of the feline decay-accelerating factor (fDAF) protein aligned with DAFs from other species. (A) Comparison of the whole amino acid sequence of DAFs. The glycosylphosphatidylinositol (GPI) anchor domains are marked with red letters. Asterisks indicate stop codons. Amino acid residues that are conserved in at least two of the four sequences are shaded, and amino acid residues identical among all four proteins are shown in black. *Legend*: hDAF, human DAF; mDAF, mouse DAF; sDAF, swine DAF.

To create pig organs that express fDAF to ensure that they are resistant to the complement in the recipient feline body, it was necessary to obtain the *fDAF* sequence. Only a partial sequence of *fDAF* was known at the beginning of this study; this sequence contained the start codon but not the 3′ part, such as the stop codon. To isolate full-length *fDAF*, we performed 3′ RACE using leukocyte cDNA from a domestic cat. The full-length *fDAF* was 1134 bp from the start codon to the stop codon. Based on the amino acid sequence, a GPI anchor domain was predicted at 1054–1056 bp, as detected in the DAF proteins of other species (Fig. 1, red letters). Comparison of the whole amino acid sequence of fDAF with that of other mammals revealed 58%, 46%, and 54% homology with the human, mouse, and pig sequences, respectively. When we compared the C-terminal amino acid sequence of fDAF with those of humans, mice, and pigs, we found that swine and feline DAFs are three amino acid residues shorter than the human and mouse DAFs (Fig. 1). We could not identify any other splicing variants of *fDAF* using 3′ RACE in this study.

### Establishment of an sEC clone expressing fDAF

**Fig 2:**
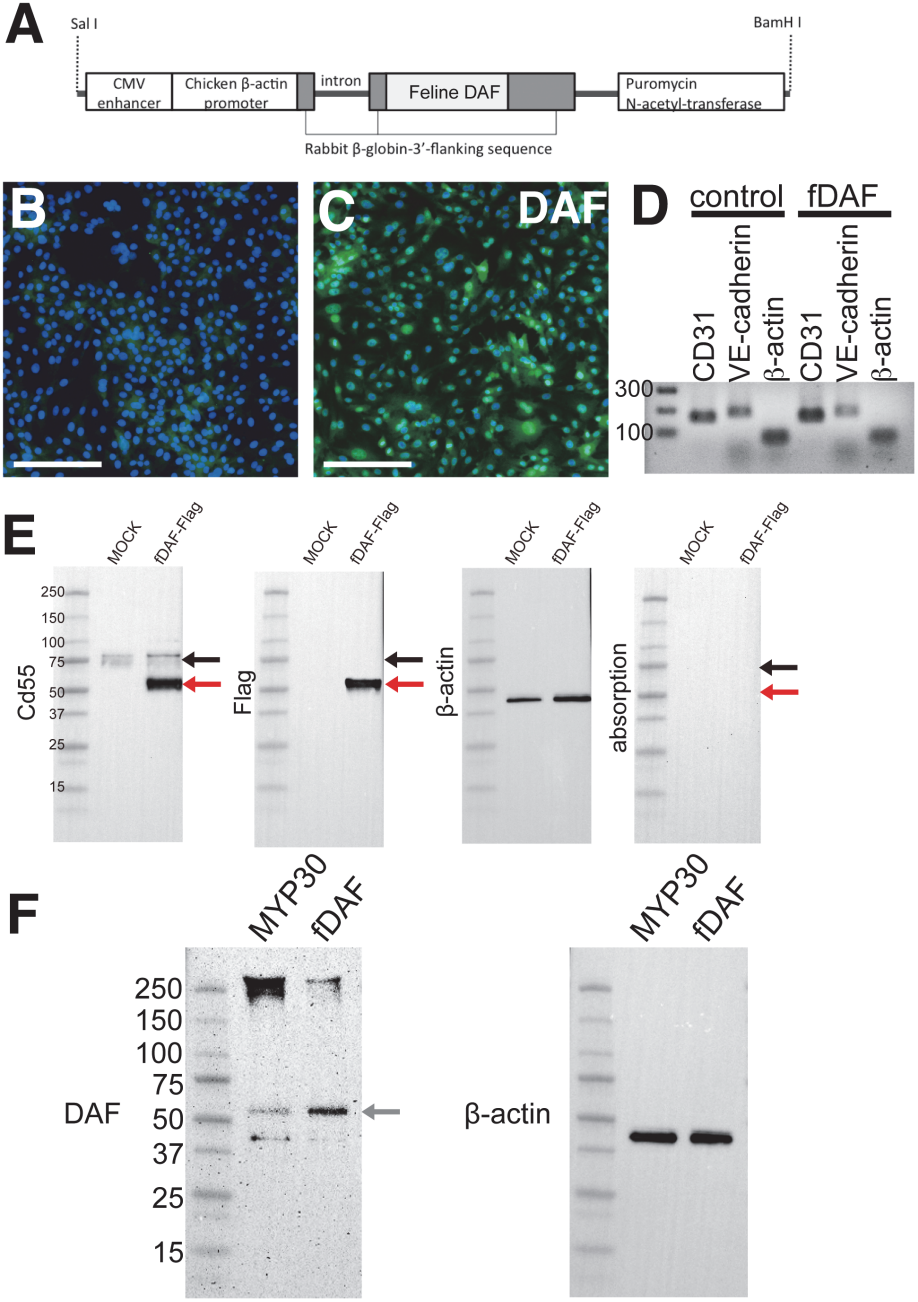
Establishment of the feline decay-accelerating factor (fDAF)-expressing swine endothelial cell (sEC) line. (A) Schematic representation of the *fDAF* expression vector between the *Sal*I and *Bam*HI sites, containing a cytomegalovirus (CMV) enhancer, a chicken β-actin promoter, *fDAF*, and the puromycin N-acetyl-transferase gene. (B and C) Immunofluorescent staining of DAF (green) in the MYP30 sEC line, with nuclear counterstaining (blue). The control MYP30 sEC line (B) and *fDAF*-expressing sEC clone (C) are also shown. Scale bars are 100 m. (D) RT-PCR analysis of endothelial cell makers (CD31 and VE-cadherin) and a house-keeping gene (ß-actin) of both the control MYP30 sEC line (control) and the *fDAF*-expressing sEC clone (fDAF). DNA maker (DNA 1kbplus ladder, invitrogen) is shown in the far left hand side. (E) Western blot analysis with an anti-DAF antibody, anti-FLAG antibody and anti-β-actin antibody of the mock control and fDAF-FLAG-transfected HEK-293 cells. The most right panel was absorption assay of anti-DAF antibody. The leftmost lane shows protein molecular weight standards in kDa. The red arrow indicates expression of fDAF-FLAG protein and the black arrow indicates endogenous human DAF expression in HEK-293 cell line. (F) Western blot analysis of the DAF protein in the MYP30 sEC line. Both the control sEC line and *fDAF*-expressing sEC clone were bound to an anti-DAF antibody (left panel) and an anti-β-actin antibody (right panel). The gray arrow indicates fDAF and sDAF expression. The leftmost lane shows protein molecular weight standards in kDa. Other bands indicate endogenous sDAF expression in the MYP30 sEC line.

To establish an fDAF-expressing swine cell clone, we used MYP30, an sEC line, which was established by Miyagawa [24]. We made this choice because hyperacute rejection mostly occurs at the level of endothelial cells in blood vessels [28]. The sEC line was transfected with *fDAF*, and cells with integration of *fDAF* into genomic DNA were selected with puromycin (Fig. 2A). This fDAF-transfected sEC clone did not exhibit any changes in the cell shape, proliferation, and adhesion (data not shown). We also investigated whether the characteristics of the sEC line are different in the fDAF-expressing sEC clone by RT-PCR of the endothelial cells maker genes CD31 and VE-cadherin [29]. We observed CD31 and VE-cadherin expression in both MYP30 sEC line and fDAF-expressing sEC clone, and their expressions were almost the same. These results indicate that fDAF-transfected sEC clone did not change its characteristics (Fig. 2D). We used immunohistochemical analysis to determine whether the established cell clone expressed fDAF (Figs. 2B, C). We observed strong expression of DAF in the *fDAF*-transfected cell line (Fig. 2C). The fluorescence intensity was not uniform among the cells, but most cells expressed DAF. We also observed very weak fluorescence in the control cells (Fig. 2B). Nonspecific binding of antibodies during immunohistochemical analysis was suspected, and we checked specificity of the anti-DAF antibody using tagged fDAF (fDAF-FLAG) in HEK-293 cells. We could detect approximately a 52-kDa band in the fDAF-expressing cells that was stained with the anti-FLAG antibody (Fig. 2E, red arrow). These results show that this antibody could detect transfected fDAF protein. In addition, we observed several proteins approximately 70–80 kDa both in the mock control and the fDAF-FLAG-expressing cells (Fig. 2E, black arrow). It has already been reported that DAF is expressed in various organs such as kidneys, lungs, spleen, testes, and blood vessels, and also the molecular weight of the protein differs among organs due to splicing variants and glycosylation [30,31]. In the human stomach cancer cell line, 70 kDa and 82 kDa variants of the CD55 protein have been reported [32], and we suspected that the band detected in both Mock and fDAF-expressing sEC cells may be the human DAF protein. We tested whether this was nonspecific staining or intrinsic expression of human DAF (hDAF) in HEK-293 cells. An absorption assay was performed to this end, and no protein bands were observed, implying that our antibody was specific (Fig. 2E). These results suggested that the extra band around 70–80 kDa may be hDAF because we confirmed that there was no nonspecific binding by the antibody in question. These results also suggest that the weak fluorescence observed in the MYP30 sEC line (Fig. 2B) may be the endogenous swine DAF (sDAF). We measured *sDAF* expression by RT-PCR and found a weak signal in the control and *fDAF*-expressing cells (data not shown). The *fDAF* mRNA was detected only in the fDAF-expressing sEC clone (data not shown). We also tried to measure the fDAF protein expression level by western blotting and found that DAF expression in the fDAF-expressing sEC clone was approximately 3-fold greater than that in the control sEC line (Fig. 2F). We also detected a band over 250 kDa and a 40-kDa band in both the MYP30 sEC line and the fDAF-expressing sEC clone, which may indicate the presence of sDAF. This follows previously reported results, which showed that several bands are observed in blood samples [30] (Fig. 2F). This result indicated that there was a strong expression of fDAF in the fDAF-transfected sEC line. These data suggested that we successfully established an fDAF-expressing sEC clone.

### *fDAF*-transfected sEC clone is resistant to feline serum

**Fig 3:**
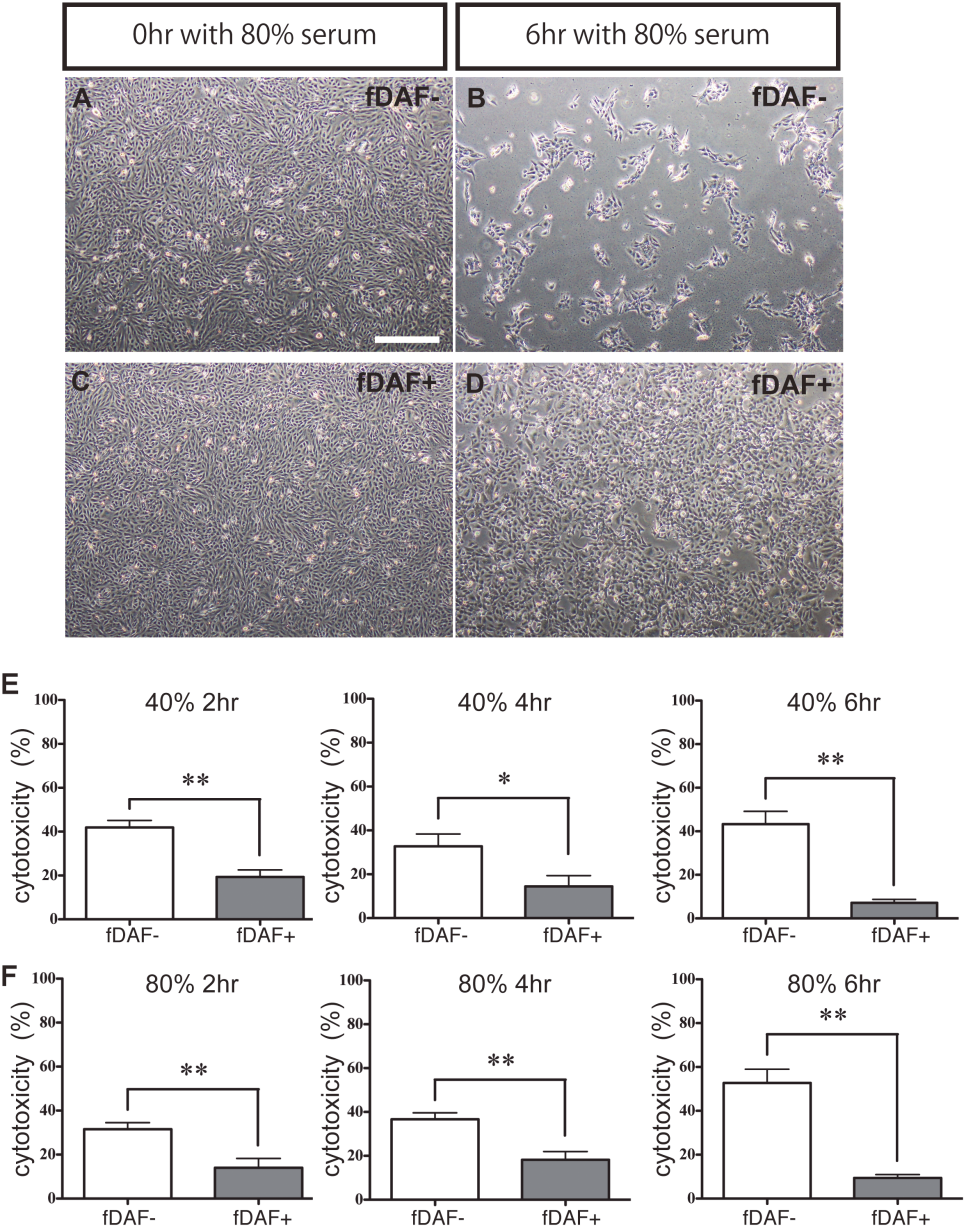
Analysis of the toxic effects of feline serum on feline DAF (fDAF)-expressing cells. (A–D) Control swine endothelial cell (sEC) line (fDAF^−^) or fDAF-expressing sEC clone (fDAF^+^) incubated with 80% feline serum. Time zero (0 h) of incubation of the control sEC line (A) and of the fDAF-expressing sEC clone (C). After a 6-h incubation, cells of the control sEC line appear small, round, and detached from the dish (B), whereas cells of the fDAF-expressing sEC clone remain attached to the dish (D). (E and F) A lactate dehydrogenase (LDH) assay of the control cells (fDAF^−^, white box) and the fDAF-expressing sEC clone (fDAF^+^, gray box) incubated with 40% feline serum (E) or 80% feline serum (F). Each data point represents mean ± SEM of five independent experiments (*P < 0.05; **P < 0.01).

We verified that fDAF conferred resistance to the cytotoxic effects of feline serum, which cause hyperacute rejection of a transplanted organ. We exposed the control cells and fDAF-expressing sEC clone to 80% feline serum (Fig. 3A, C; n = 5). When exposed to the 80% feline serum for 6 h, cells of the control sEC line were small, round, and floating, whereas cells of the fDAF-expressing sEC clone were affected negligibly by the feline serum, and most cells remained attached to the culture dish (Fig. 3B, D). These results suggested that fDAF conferred resistance to feline serum on sECs. Because acute rejection starts within minutes to hours [11,33,34], we measured the cytotoxic effects of feline serum using an LDH assay 2, 4, and 6 h after addition of 40% (low concentration) or 80% (high concentration) feline serum to the culture medium (Figs. 3E, 3F; n = 5 for each experiment). At these time points, feline serum was always toxic to the control sEC line at both the high and low concentration (Figs. 3E, F). In contrast, feline serum was less toxic to the fDAF-expressing sEC clone even when the feline serum concentration was high. Measured cytotoxicity in the control sEC line in 40% feline serum was 42%, 33%, and 43% and for 80% feline serum, the cytotoxicity was 32%, 37%, and 40% at 2, 4, and 6 h respectively. Measured cytotoxicity in the fDAF-expressing sEC clone in 40% feline serum was 19%, 14%, and 7% and in 80% feline serum, the cytotoxicity was 14%, 18%, and 9% at 2, 4, and 6 h respectively (Fig. 3E, F).

We compared the cytotoxicity in the control sEC line and the fDAF-expressing sEC clone; at 2 h, the cytotoxicity in the fDAF-expressing sEC clone was approximately half of that in the control sEC line, and this difference increased at later time points (Fig. 3E, F). Moreover, we confirmed that the resistance to the cytotoxicity of feline serum persisted at later time points (8, 12, and 24 h). At 8 h, the same resistance was observed as at 6 h. Cells of the fDAF-expressing sEC clone were still attached to the dish after 12 and 24 h of culture; however, the cytotoxicity could no longer be measured using the LDH assay (data not shown).

These results indicated that fDAF conferred resistance to the cytotoxicity of feline serum on sECs.

## Discussion

Regenerative medicine is a new discipline, and clinical studies using stem cells from patients for the treatment of age-related macular degeneration are in progress in Japan. Nonetheless, generation of complex organs, such as the kidney, is difficult because the kidney consists of many cell types, including glomerular podocytes, endothelial cells, mesangial cells, interstitial cells, tubular epithelial cells, and connecting duct cells. De novo reconstruction of a kidney from these cells is very difficult, and we previously tested kidney reconstruction using a developing heterozoic embryo as an organ factory [1,2]. We also reported a neokidney derived from human MSCs in a developing rat embryo [1,2,10,35]. These experiments indicate that the de novo generation of a complete kidney is possible. The neokidney produces urine, secretes human EPO, and physiologically shifts the plasma EPO concentration to a normal level in an anemic host animal [3]. These findings are promising because this neokidney could be used for the treatment of CKD patients and pets to reduce anemia. Many domestic cats suffer from CKD, and EPO can improve CKD-induced anemia. However, cats produce antibodies against EPO, even feline EPO. Therefore, this problem may be addressed by transplanting an EPO-producing neokidney (derived from MSCs) from the cat with CKD into a developing pig kidney. This neokidney may secrete host cat’s EPO (without causing an immune response) and thereby improve the CKD-induced anemia in the host cat.

Nevertheless, portions of this neokidney, including the endothelial cells, are derived from swine cells, causing its rejection in the host cat. Many transplantation therapies involve immunosuppressive treatment to prevent rejection. Although immunosuppressive agents are also effective for transplantation therapies in cats, anti-pig antibodies frequently trigger hyperacute rejection before the immunosuppressive agent starts to have its effects. In xenotransplantation procedures between humans and pigs, human DAF-expressing swine cells have been successfully used to prevent hyperacute rejection [36]. Therefore, the same strategy could be utilized for transplantation between pigs and cats. In this study, we demonstrate that fDAF-expressing sECs are resistant to hyperacute rejection caused by feline serum.

The *fDAF* sequence was determined using the 3′ RACE method. We found a *fDAF* sequence registered in the NCBI database (XP_003999494), but that sequence is longer than ours and has a different 3′ region. We tried but could not clone this longer cDNA in this study. The longer *fDAF* sequence was predicted by automated computational analysis; therefore, it may not exist or may be a splicing variant that we did not have in this study. We also attempted to confirm the sequence in the Ensemble database using 5′ RACE, but this experiment was unsuccessful because the 5′ region is GC rich. Clearer genomic data will help to characterize fDAF mRNA more completely.

Using our cloned *fDAF* sequence, we found that hDAF is more similar to fDAF than to DAFs from other species. The amino acid residues at the 3′ end differ between feline and swine DAFs and between human and mouse DAFs, whereas the 3′ sequence in DAF of the giant panda is identical to that of fDAF.

Analysis of an established fDAF-transfected sEC clone showed that fluorescence intensity is not uniform when we probe fDAF using an anti-DAF antibody (Fig. 2C). We also found that weak expression of sDAF is present in the MYP30 sEC line (control cells), according to immunohistochemical analysis (Fig. 2B) and western blotting (Fig. 2F). Most probably, nonuniform fluorescence was detected because nonuniform expression of sDAF and uniform expression of fDAF were overlapping. In the western blot analysis of the control sEC line and fDAF-expressing sEC clone, we observed several protein bands on the membrane (Fig. 2F). These bands matched that of the isoforms of sDAF previously reported [30]. In Fig. 2E, we detected the hDAF protein in the 70–80-kDa bands, and fDAF in the 52-kDa band. Human and feline DAF are highly similar in their amino acid sequence; however, Western blot analysis showed that they have different sizes. This may be due to the presence of splicing variants of hDAF. In NCBI, human DAF is described has presenting splicing variants (NM_000574 and NM_001114752) and a long type (NM_001114752) approximately 60 amino acid longer than the general form (NM_000574). Here we did not check which splicing variant was present in the HEK-293, but from our absorption study, the 70–80-kDa band disappeared suggesting that the 70–80 kDa protein is in fact hDAF.

Our fDAF-expressing sEC clone shows resistance to feline serum. Using an LDH assay, we showed significant differences in the cytotoxic effects on the control and fDAF-expressing cells after 2 h of incubation with feline serum. Our results also revealed that fDAF-expressing cells continue to exert resistance with longer incubation periods (Fig. 3). In contrast, in the control cells, cytotoxicity increased with time. We also checked cytotoxicity at 12 and 24 h. At these time points, the LDH assay could not be performed, but we saw that the fDAF-expressing cells were still attached to the culture dish (data not shown). These results indicate that fDAF causes resistance to hyperacute rejection for significant periods (at least 24 h), and this effect may be useful for xenotransplantation-related therapies.

Furthermore, when we increased the feline serum concentration to 80%, the differences between fDAF-expressing and control cells persisted. These results suggest that our cloned fDAF is functional in swine cells and confers strong resistance to hyperacute rejection caused by feline serum. Therefore, these “felinized” swine cells may not undergo hyperacute rejection after xenotransplantation. Moreover, the method for fDAF expression in swine cells may become a useful tool for xenotransplantation therapies. During xenotransplantation between humans and other mammalian species such as pigs, there is a problem of the α-galactosyl (α-Gal) epitope. Only humans and higher primates lack α-Gal among mammals, and the α-Gal epitope from other species causes hyperacute rejection in humans after xenotransplantation [37,38]. Thus, for successful xenotransplantation between humans and pigs, one of the α-Gal-related genes should be inactivated in the swine cells. In contrast, both cats and pigs have the α-Gal-related genes; thus, the α-Gal epitope may not cause hyperacute rejection in the donor. For this reason, it is believed that a xenotransplant between a cat and a pig has a better chance of success than that between a human and a pig.

Our next challenge is to create a felinized transgenic pig and a neokidney using MSCs from a cat with CKD. In the future, our research may facilitate treatment of pet cats with CKD.
